# Icaritin Promotes Myelination by Simultaneously Enhancing the Proliferation and Differentiation of Oligodendrocyte Precursor Cells

**DOI:** 10.3390/molecules28155837

**Published:** 2023-08-03

**Authors:** Feifei Yang, Han Wen, Siqi Ma, Qi Chang, Ruile Pan, Xinmin Liu, Yonghong Liao

**Affiliations:** 1 Key Laboratory of Bioactive Substances and Resources Utilization of Chinese Herbal Medicine (Ministry of Education), Institute of Medicinal Plant Development (IMPLAD), Chinese Academy of Medical Sciences & Peking Union Medical College, No. 151 Malianwa North Road, Haidian District, Beijing 100193, China; ffyang@implad.ac.cn (F.Y.); hwenimplad@163.com (H.W.); sqma@implad.ac.cn (S.M.); qchang@implad.ac.cn (Q.C.); rlpan@implad.ac.cn (R.P.); 2 Institute of Drug Discovery Technology, Ningbo University, No. 818 Fenghua Road, Ningbo 315211, China; liuxinmin@hotmail.com

**Keywords:** demyelination, oligodendrocyte precursor cells differentiation, proliferation, icaritin, traditional Chinese medicine

## Abstract

Myelin repair, which is known as remyelination, is critical to the treatment of neurodegenerative diseases, and myelination depends on not only the differentiation of oligodendrocyte precursor cells toward oligodendrocytes but also the renewal of oligodendrocyte precursor cells under pathological conditions. However, simultaneously promoting the differentiation and proliferation of oligodendrocyte precursor cells in lesions remains an unmet challenge and might affect demyelinating diseases. Kidney-tonifying herbs of traditional Chinese medicine (TCM) are effective in improving the symptoms of degenerative patients. However, herbs or compounds with dual functions are unverified. The purpose of this study was to find a kidney-tonifying TCM that synchronously improved the differentiation and proliferation of oligodendrocyte precursor cells under pathological conditions. Compounds with dual functions were screened from highly frequently used kidney-tonifying TCM, and the effects of the obtained compound on remyelination were investigated in an in vitro oligodendrocyte precursor cell differentiation model under pathological conditions and in demyelinating mice in vivo. The compound icaritin, which is an active component of Yin-Yang-Huo (the leaves of *Epimedium brevicornu* Maxim), demonstrated multiple effects on the remyelination process, including enhancing oligodendrocyte precursor cell proliferation, facilitating the differentiation of neural progenitor cells toward oligodendrocyte precursor cells and further toward oligodendrocytes, and maturation of oligodendrocytes under corticosterone- or glutamate-induced pathological conditions. Importantly, icaritin effectively rescued behavioral functions and increased the formation of myelin in a cuprizone-induced demyelination mouse model. The multiple effects of icaritin make it a promising lead compound for remyelination therapy.

## 1. Introduction

Myelin sheaths are important in neural signal transport, and damage or loss of myelin (demyelination) causes neurodegenerative diseases such as multiple sclerosis (MS) and Alzheimer’s disease (AD) [1]. Remyelination is the self-healing process for demyelination in the central nervous system (CNS) [2]; however, this ability declines with aging or under pathological conditions and cannot replenish damaged myelin completely [3]. Therefore, it is necessary to elevate remyelination to restore CNS function. The formation of myelin depends on the maturation of oligodendrocytes, which originate from oligodendrocyte precursor cells in the CNS, but oligodendrocytes cannot migrate or proliferate, which impairs myelin generation [4]. Therefore, promoting the proliferation of oligodendrocyte precursor cells and facilitating the differentiation of oligodendrocyte precursor cells toward oligodendrocytes are critical for remyelination and neurodegenerative disease therapy.

Current therapeutic regimens for degenerative diseases are aimed at decreasing relapse or postponing disability using monoclonal antibodies or immunosuppressive agents, and symptoms are mitigated rather than cured in response to these treatments [5]. To the best of our knowledge, there is no medicine that can reverse myelin damage or loss. An important reason for the failure of opicinumad (BIIB033, anti-LINGO-1 antibody) in phase II clinical trial was the deficiency of oligodendrocyte precursor cells in the lesions of old patients under pathological conditions [6,7], which was neglected in a previous study. Critical pathological characteristics of demyelination are increased levels of glutamate (Glu) and corticosterone (CORT) [8], and overexpressed Glu induces excitotoxicity and axonal apoptosis in the brain [9], while increased CORT inhibits the proliferation of oligodendrocyte precursor cells [10]. Therefore, compounds that restore the neuronal damage induced by Glu and CORT are ideal candidates for remyelination.

The kidney-tonifying herbs of traditional Chinese medicine (TCM) are effective in improving the cognition and motor functions of degenerative patients [11,12]. The kidney is closely related to the function of the CNS; the brain is a sea of marrow, and kidney deficiency is the main cause of neurodegeneration in Chinese medicine theory. Nourishing the kidneys benefits CNS diseases. The effect of kidney-tonifying formulas on degenerative diseases has been well documented. One of the well-known kidney-tonifying formulations is BaZiBuShen (No. B20020585), which has been officially approved by the State Food and Drug Administration in China (SFDA) for nourishing the kidney and protecting against aging-associated CNS diseases. Xu confirmed that an ancient kidney-tonifying formula containing Rou-Cong-Rong, Yin-Yang-Huo, and Huang-Jing exerted significant neuroprotective effects against oxidative damage [13]. Most of these kidney-tonifying formulas contain over ten herbs, and certain herbs are frequently used, such as Rou-Cong-Rong and Yin-Yang-Huo; however, which of these kidney-tonifying herbs and which component of these herbs is efficient in remyelination and how they affect the formation of myelin are largely unknown. Icaritin, an active flavonoid from one of the most frequently used herbs in the kidney-tonifying formula Yin-Yang-Huo (*Epimedium brevicornu* Maxim), is reported to exert neuroprotection and benefit myelin formation. Icaritin activates the Nrf2/Keap1 signaling pathway for oxidative stress relief [14] and alleviates glutamate-induced neuronal damage by inactivating GluN2B-containing NMDARs through the ERK/DAPK pathway, which are critical for myelin formation under pathological conditions [15]. The effects of icaritin on the myelination process have not been systematically studied. In this study, we screened promoters of myelination from frequently used herbs in kidney-tonifying formulas and systematically investigated their effects on oligodendrocyte precursor cell survival and differentiation under pathological conditions.

## 2. Results and Discussion

### 2.1. Icaritin Promotes Oligodendrocyte Precursor Cell Viability and Differentiation

**Scheme 1:**
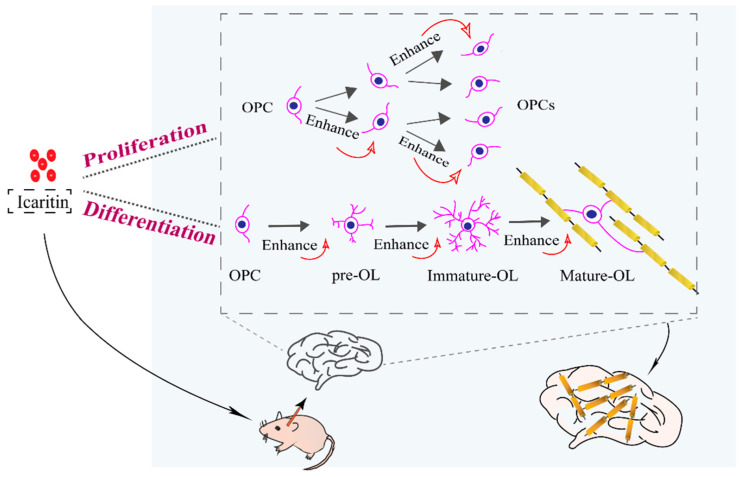
Schematic illustration of icaritin in synchronizing oligodendrocyte precursor cell (OPC) proliferation and differentiation. OL: oligodendrocyte.

Myelination in the CNS depends on the differentiation of oligodendrocyte precursor cells into oligodendrocytes. Efforts have been made to screen promoters of differentiation in vitro. However, the oligodendrocyte precursor cell pool is depleted under chronic degeneration; consequently, oligodendrocyte precursor cells in the lesion are deficient and hinder remyelination. This factor in the failure of remyelination has been neglected in previous studies [8] (Scheme 1). In TCM theory, the kidney yin deficiency is a critical factor in demyelination, and the classical treatment for demyelinating diseases was the administration of kidney-tonifying herbs [11].

**Figure 1:**
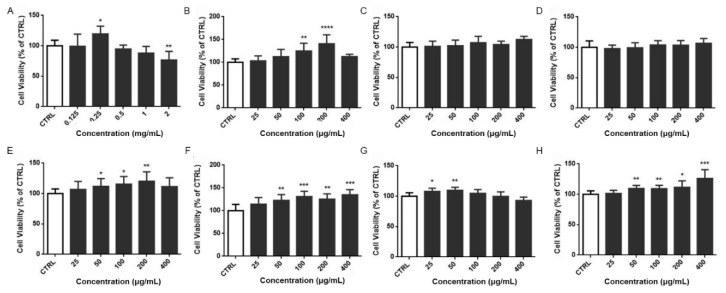
Neuroprotective effects of ethanol extracts from kidney-tonifying herbs on oligodendrocyte precursor cells. Lu-Rong (**A**), Yin-Yang-Huo (**B**), Zhi-Zi (**C**), Di-Huang (**D**), Shu Di-Huang (**E**), Huang-Qi (**F**), She-Chuang-Zi (**G**), Yuan-Zhi (**H**). * *p* < 0.05; ** *p* < 0.01; *** *p* < 0.001; **** *p* < 0.0001, compared with CTRL group.

In this study, we screened eight frequently used kidney-tonifying TCMs to obtain herbs with protective effects on oligodendrocyte precursor cells using the MTT assay. As shown in Figure 1, the ethanol extracts of the six raw materials, namely Lu-Rong, Yin-Yang-Huo, Di-Huang, Huang-Qi, She-Chuang-Zi, and Jie-Geng, promoted the viability of oligodendrocyte precursor cells at the experimental concentrations. A significantly higher viability than CTRL indicated the enhancement of proliferation.

The formation of myelin is a multistep process, including the differentiation of neural progenitor cells to oligodendrocyte precursor cells and oligodendrocytes, the maturation of oligodendrocytes, and the formation of myelin. Here, we investigated the effects of the active components (dose screening is shown in Supporting Figure S1) on the differentiation of neural progenitor cells toward A2B5^+^ and PDGFα^+^ positive oligodendrocyte precursor cells (Table 1, Supporting Figure S2). As shown in Table 1, A2B5^+^ oligodendrocyte precursor cells were 61.53 ± 1.89% in the control group, significantly increased levels of A2B5^+^ cells were found in the catalpol (*p* = 0.0061)-, geniposide (*p* = 0.0197)- and icaritin (*p* = 0.0003)-treated cells, and the ratio reached 70.83 ± 1.60%, 69.20 ± 3.66% and 75.34 ± 2.97%, respectively. This result confirmed the effects of these compounds on promoting the differentiation of neural progenitor cells toward oligodendrocyte precursor cells. Similarly, MBP^+^ oligodendrocytes were examined to evaluate the differentiation of oligodendrocyte precursor cells to oligodendrocytes (Table 2). We found that the positive control polyornithine, 3,3,5-Triiodo-l-thyronine (T3) significantly enhanced the proportion of MBP^+^ cells (*p* = 0.0028). Icaritin and icariin could promote the differentiation of oligodendrocyte precursor cells toward oligodendrocytes. Overall, icaritin affected both differentiation stages.

**Figure 2:**
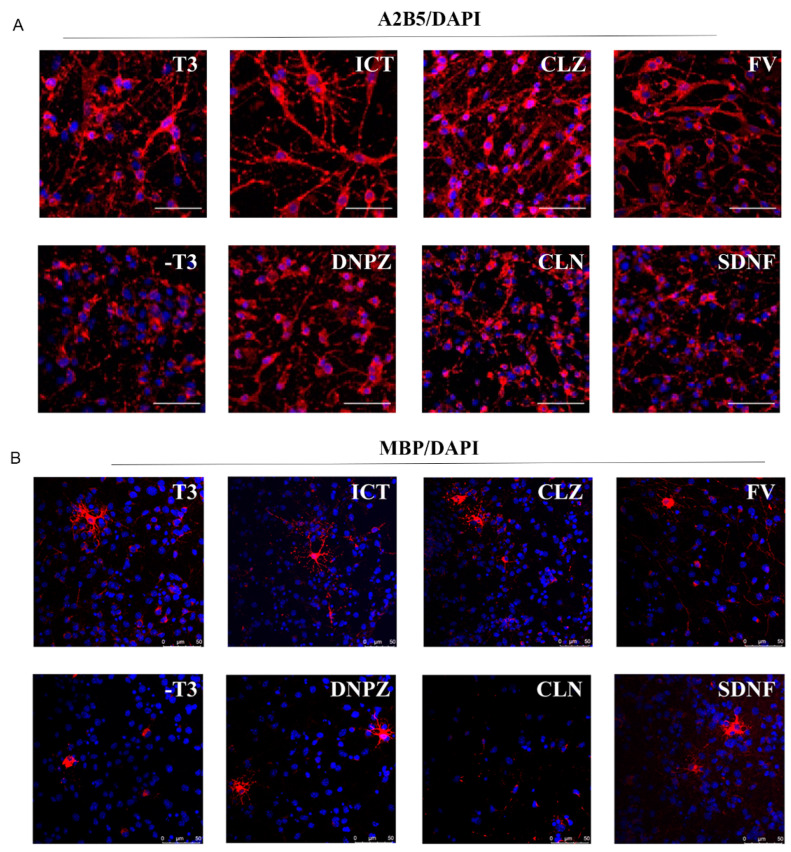
ICT promoted the differentiation of oligodendroglial lineage. (**A**) Representative images of A2B5^+^ oligodendrocyte precursor cells (red) after coincubation with ICT or other small molecules for 72 h (5 μM), scale bar represents 50 μm. (**B**) Representative images of MBP^+^ oligodendrocytes (red) after coincubation with ICT or other small molecules for 72 h (5 μM). Nucleus were stained with DAPI (blue). T3: 3,3,5-Triiodo-l-thyronine; ICT: icaritin; CLZ: clozapine; FV: fulvestrant; DNPZ: donepezil; CLN: clemastine; SDNF: sildenafil.

Small molecules, such as fulvestrant (FV), clemastine (CLN), clozapine (CLZ), donepezil (DNPZ), and sildenafil (SDNF), have been reported to be effective in promoting oligodendrocyte differentiation and remyelination. Donepezil has been approved to treat Alzheimer’s disease and can promote oligodendrocyte generation and remyelination [16]. In this study, these small molecules were used as controls to evaluate the effect of icaritin on the differentiation and maturation of oligodendrocytes [17]. As shown in Figure 2A, cells treated with T3 and icaritin showed bi- or tribranching morphology, indicating the maturation of oligodendrocytes [18]. In contrast, no such multibranching morphology was observed in cells without T3 or in those treated with other small molecules. To verify the formation of mature oligodendrocytes, MBP was stained. Cells treated with icaritin or small molecules exhibited mature oligodendrocyte morphology with multiple soma, which was not seen in cells without T3 or CLN treatment (Figure 2B). Collectively, icaritin promotes the differentiation of neural progenitor cells into oligodendrocyte precursor cells and oligodendrocytes, as well as the maturation of oligodendrocytes.

### 2.2. Icaritin Promotes Oligodendrocyte Precursor Cell Proliferation under Pathological Conditions In Vitro

**Figure 3:**
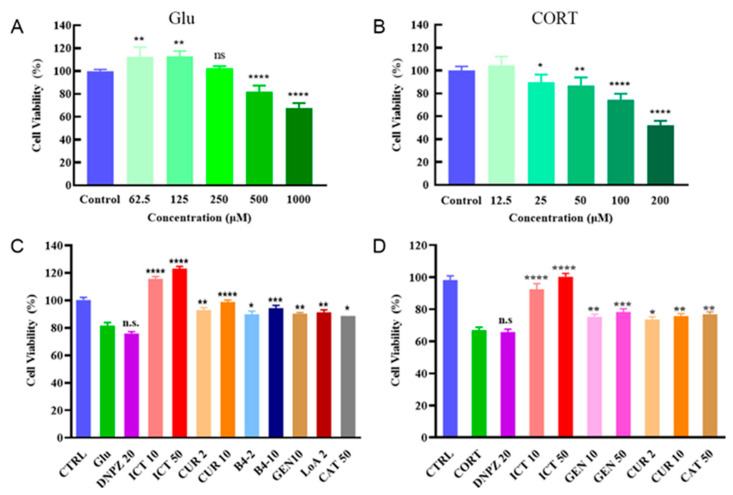
ICT protects oligodendrocyte precursor cells under pathological conditions. The cell viability of neural progenitor cells at different concentrations of Glu (**A**) and CORT (**B**). Protection of TCM constituents under Glu (500 μM, **C**) or CORT (100 μM, **D**) induced pathological conditions. Data are presented as mean ± SD. *n* = 6. ns indicates no significant difference (*p* > 0.05); * *p* < 0.05; ** *p* < 0.01; *** *p* < 0.001; **** *p* < 0.0001 compared with control (**A**,**B**); n.s. indicates no significant difference (*p* > 0.05); * *p* < 0.05; ** *p* < 0.01; *** *p* < 0.001; **** *p* < 0.0001 compared with Glu or CORT groups (**C**,**D**). ICT: icaritin; Glu: glutamate; CORT: corticosrone.

Active compounds are usually phenotypically screened using oligodendrocyte cellular models under normal culture conditions rather than a simulated pathological environment, including donepezil. However, high levels of Glu and CORT can be detrimental to the proliferation of oligodendrocyte precursor cells under neurodegenerative conditions [19]. In this study, we took the properties of the neurodegenerative microenvironment into consideration. Glu and CORT could induce cell damage in a concentration-dependent manner, and the presence of Glu at a concentration of 500 μM and CORT at 100 μM resulted in moderate decreases in cell viability to 81.56 ± 2.23% and 67.01 ± 1.86%, respectively (Figure 3A,B). In both Glu- and CORT-induced models, donepezil did not improve the viability of oligodendrocyte precursor cells at a concentration of 20 μM (Figure 3C,D, Supporting Tables S1 and S2), and cell viability significantly decreased to 48.67% in response to a concentration of 100 μM (Supporting Table S1). In contrast, the addition of icaritin (10 or 50 μM) effectively reversed the cell damage induced by Glu and CORT and promoted proliferation (Figure 3C,D), confirming that icaritin exerted neuroprotection against CORT- and Glu-related damage, which is critical for oligodendrocyte precursor cells recruited in lesions.

**Figure 4:**
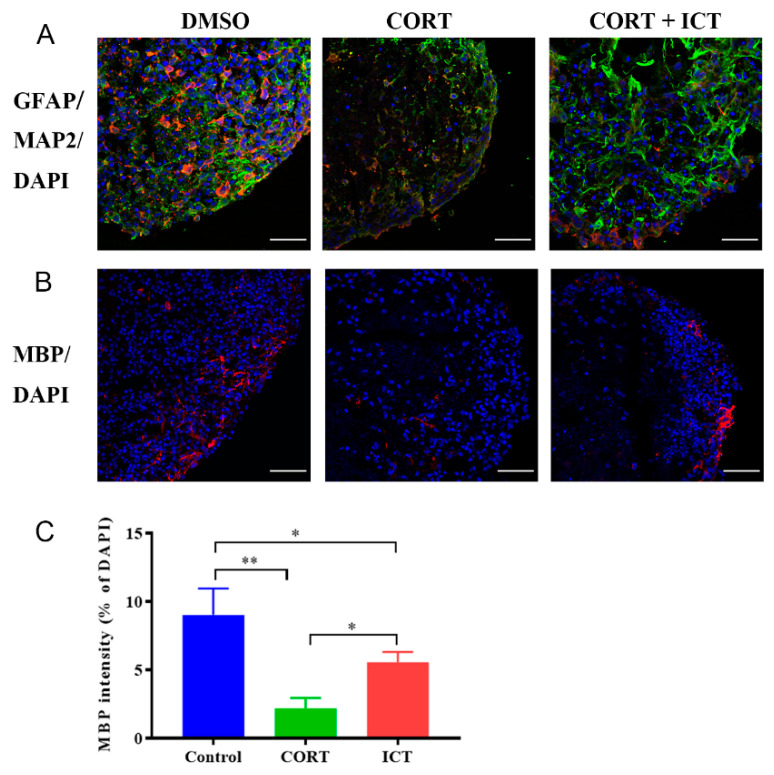
ICT promoted the differentiation of mouse neurospheres under pathological conditions. (**A**) ICT promoted the differentiation of mouse neurospheres toward GFAP^+^ astrocytes (green) and inhibited the differentiation toward MAP2^+^ neurons (red) in LXY30-alginate hydrogel on Day 30. Scale bar represents 50 μm. (**B**) ICT promoted the differentiation of mouse neurospheres toward MBP^+^ oligodendrocytes (red) in LXY30-alginate hydrogel on Day 60. Scale bar represents 50 μm. * *p* < 0.05, ** *p* < 0.01. (**C**) Quantification analysis for MBP^+^ area on day 60 (Data are presented as mean ± SEM. *n* = 3). ICT: icaritin.

The effects of icaritin on the differentiation of neural progenitor cells toward oligodendrocytes under pathological conditions were evaluated using an in vitro 3D hydrogel, which is suitable for the long-term culture of neural progenitor cells [16]. Differentiation status was analyzed on Day 30 and Day 60, and MAP2, GFAP and MBP were stained to characterize neurons, astrocytes, and oligodendrocytes, respectively. On Day 30, cells treated with CORT exhibited incomplete morphology and downregulated expression of GFAP and MAP2, whereas coincubation with icaritin promoted the differentiation of neural progenitor cells (Figure 4A), which is in accordance with cells under normal culture conditions [20]. On Day 60, the expression of MBP was significantly decreased in CORT-treated cells compared with DMSO-treated cells (*p* = 0.0014), confirming the inhibition of differentiation toward oligodendrocytes due to CORT (Figure 4B). In contrast, the expression of MBP significantly increased after coincubation with icaritin (*p* = 0.0407, Figure 4C). Overall, these results confirmed that icaritin played dual roles in remyelination under pathological conditions: protecting cells from neurodamage, promoting the differentiation of neural progenitor cells toward oligodendrocytes.

### 2.3. Icaritin Improved Spontaneous Activity and Remyelination In Vivo

**Figure 5:**
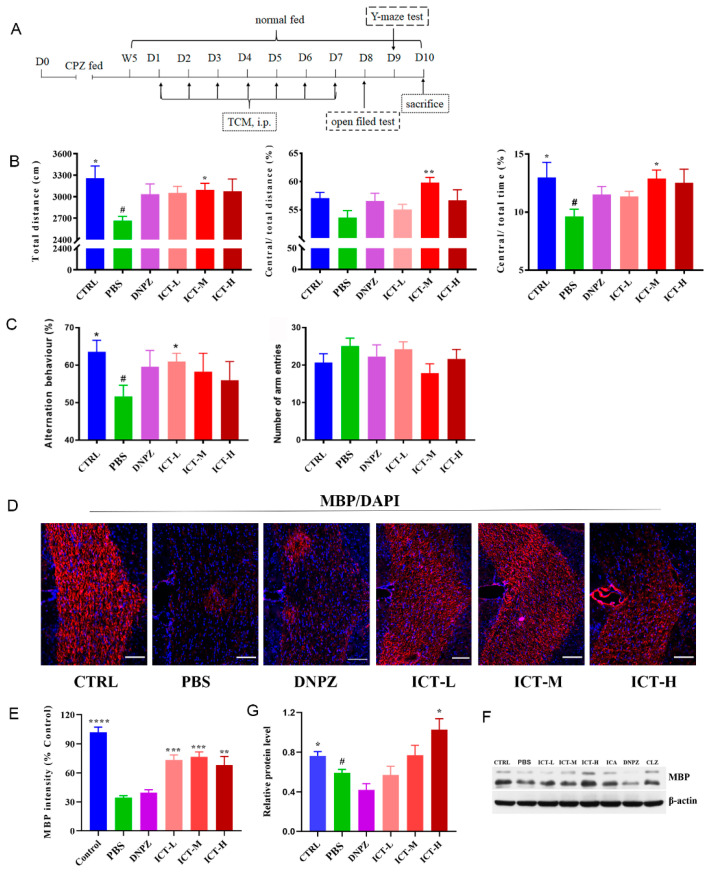
ICT improved motor function and enhanced remyelination in vivo in demyelinating mice. (**A**) Scheme of CPZ-induced demyelinating mice model development and regimens. (**B**) ICT improved the locomotor activities of demyelinating mice in the total distance (left), Central/Total distance (middle) and Central/Total time (right) in an open field test, *n* = 14. * *p* < 0.05, ** *p* < 0.01, compared with PBS (CPZ treated mice). # *p* < 0.05 compared with the CTRL group. (**C**) ICT increased the working and reference memory of demyelinating mice, *n* = 14. * *p* < 0.05 compared with PBS (CPZ-treated mice). # *p* < 0.05 compared with the CTRL group. (**D**) Representative images of MBP expression in the corpus collosum of demyelinating mice after different treatments, scale bar represents 20 μm. (**E**) Semi-quantification of MBP expressions in the corpus collosum of demyelinating mice. ** *p* < 0.01; *** *p* < 0.001; **** *p* < 0.0001 compared with PBS (CPZ treated mice). Data were presented as mean ± SEM. *n* = 3. (**F**) Expressions of MBP in the hippocampus with western- blot analysis. (**G**) The grayscale analysis of MBP expressions relative to β-actin. * *p* < 0.05, compared with PBS (CPZ treated mice). # *p* < 0.05 compared with the CTRL group. Data are presented as mean ± SEM. *n* = 3. ICT: icaritin; CPZ: cuprizone.

Cuprizone (CPZ)-induced demyelination in mice is associated with decreased spontaneous activity and cognition [21]. Donepezil, as an approved medicine for Alzheimer’s disease, was used as a positive control in the in vivo spontaneous activity test. In this study, we assessed how icaritin affected the in vivo spontaneous activity of demyelinating mice via the open field test (OFT, Figure 5A). As shown in Figure 5B, demyelinating mice showed significantly decreased total distances (*p* = 0.002, left) and percentages of central/total time (*p* = 0.0319, right), suggesting reduced spontaneous activity after demyelination. No improvement in spontaneous activity was observed in donepezil-treated mice after 1-week of therapy, which might be caused by insufficient therapeutic duration [16]. In contrast, the total motion distance (left) and the distance (and/or time) spent in the central area (middle/right) were increased after treatment with icaritin for 1-week, confirming the improvement in spontaneous activity and the restoration of motor function in demyelinating mice. Spontaneous alteration behavior is an indicator of cognition by testing the short-term spatial working memory. In this study, it was used to evaluate the effects of icaritin on the cognition of demyelinating mice. From Figure 5C, the spontaneous alteration behaviors significantly decreased in demyelinating mice compared with normal mice (*p* = 0.0185), and alternation improved in response to treatment with donepezil. The highest alteration behavior was recorded in mice treated with icaritin (*p* = 0.0303, left), although no difference was found in the total number of arm entries (right). The results confirmed stronger spatial working memory restoration after icaritin treatment than after donepezil treatment.

In addition, we investigated whether icaritin enhanced in vivo remyelination. MBP is a crucial component of total myelin proteins, and it is important to maintain the integrity of the myelin sheath in the CNS. The intensity of MBP in the corpus was analyzed by immunofluorescence and western blot. As shown in Figure 5D,E, demyelinating mice (PBS) showed a 66.30% reduction in the expression of MBP compared with normal mice (CTRL), and no notable increase in MBP expression (38.97% of the normal mice) was found in mice treated with donepezil, indicating no benefit from donepezil in this regimen. Donepezil has been shown to promote remyelination in a cuprizone-induced demyelination mouse model [16]. In that study, 2 weeks but not 1 week of treatment with donepezil could increase MBP expression. In contrast, the MBP intensity was 1.90-fold (65% of normal mice) higher than at baseline upon demyelination following 1 week of treatment with icaritin, indicating that even in a short duration of icaritin treatment, it promoted myelin restoration (Figure 5E). We further verified the enhanced MBP expression with western blot analysis (Figure 5F,G). The results showed that the MBP expression in demyelinating mice was decreased compared with that in normal mice, and donepezil-treated mice demonstrated even lower MBP expression following this regimen. The semiquantitative results in Figure 5G suggested that the expression of MBP increased in a dose-dependent manner post-icaritin treatment, indicating icaritin’s potential for promoting in vivo remyelination. The results indicated that successive administration of 20 mg/kg/day of icaritin for 7 days mitigated the CPZ-induced demyelination in mice, and the effects were dose-dependent.

### 2.4. Potential Mechanisms of Icaritin

**Figure 6:**
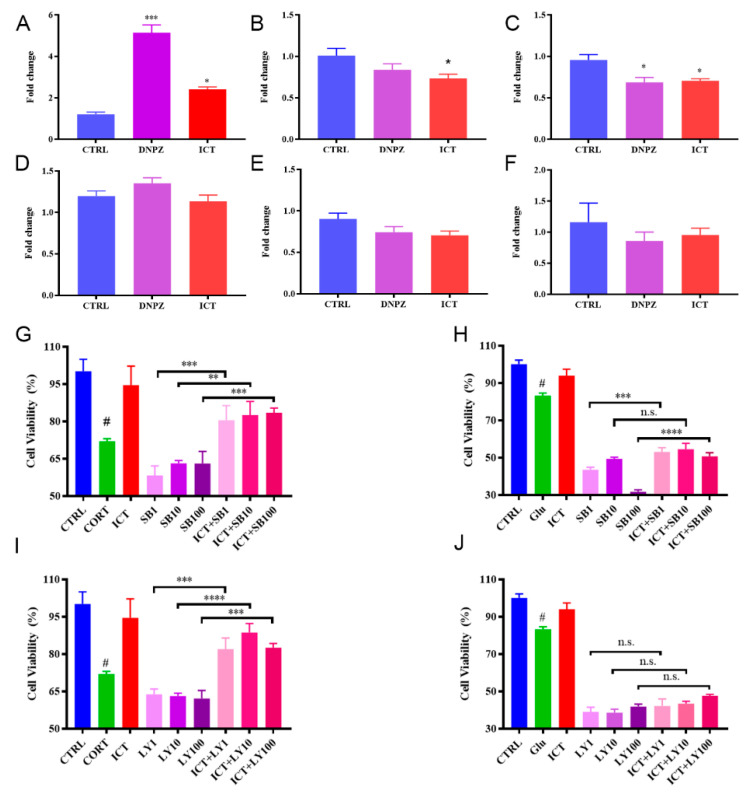
Effects of ICT on myelin-related genes and the neuroprotective effects of ICT under pathological conditions when differentiation pathways were blocked. Quantification of myelin-related genes expression after incubation with ICT or DNPZ for 48 h, MBP *(***A**), *NKX2.2 (***B**), *PDGFaa* (**C**), *Olig2 (***D**), *SOX10* (**E**) and *NGN3* (**F**). * *p* < 0.05, *** *p* < 0.001 compared with CTRL. Data are presented as mean ± SD. *n* = 4. Protective effects of ICT under CORT- induced pathological conditions when p38MAPK (**G**) or PI3K/Akt (**I**) was blocked. Mitigated protective effects of ICT under Glu- induced pathological conditions when p38MAPK (**H**) or PI3K/Akt (**J**) was blocked. Data are presented as mean ± SD. *n* = 6. n.s. indicates no significant difference (*p* > 0.05); ** *p* < 0.01; *** *p* < 0.001; **** *p* < 0.0001 compared with CORT/Glu group. # *p* < 0.05 compared with CTRL. ICT: icaritin. Glu: glutamate; CORT: corticosrone.

To explore how icaritin affects the expression of myelination-related genes, cellular expressions of MBP, *NKX2.2*, *PDGFaa*, *Olig2*, *SOX10*, and *NGN3* were analyzed by PCR after incubation with icaritin (Figure 6A–F). The expression of MBP in icaritin- and donepezil-treated cells was significantly higher than that untreated cells (*p* = 0.0263, *p* = 0.0005, respectively), indicating an increase in the proportion of oligodendrocytes (Figure 6A). The expression of *NKX2.2* was downregulated after coincubation with icaritin, which is beneficial to differentiation, since *NKX2.2* inhibited the differentiation of oligodendroglial lineage cells [22,23], while no such change was found in donepezil-treated cells. Decreased expression of *PDGF-aa* was observed in both donepezil- and icaritin-treated cells, but it may not affect differentiation at the current stage as it plays a role in later stages of differentiation [24]. No change was found in the expression of *Olig2*, *SOX10*, or *NGN3*. These results suggested that icaritin regulated specific myelin-related genes at the transcriptional level.

Mitogen-activated protein kinase (p38 MAPK) and phosphatidylinositol 3-kinase (PI3K)/protein kinase B (Akt) were reported to affect the proliferation and differentiation of oligodendrocyte precursor cells and the migration of oligodendrocytes [25,26]. In this study, SB203580 and LY294002 were used to specifically block these factors to investigate whether icaritin affects cell viability after inhibition. Significantly higher cell viability was found in cells that were coincubated with icaritin than in the inhibitor-only groups under CORT-induced pathological conditions when the p38 MAPK or PI3K/Akt pathway was inhibited (*p* = 0.0001, *p* < 0.0001, *p* < 0.0001, respectively, Figure 6G, and *p* = 0.0003, *p* < 0.0001, *p* = 0.0002, respectively, Figure 6I). For the Glu- induced pathological conditions, no protection was observed (Figure 6H,J). The results indicated that icaritin has the potential to reverse CORT-related damage under pathological conditions.

Nevertheless, there are limitations in this study that warrant further investigation. We only confirmed that icaritin promoted the differentiation of mouse neurospheres under pathological conditions; the co-incubation of icaritin with mouse neurospheres under normal conditions would supplement the role of icaritin in differentiation. In addition, how icaritin affects the behavior of control mice is desirable to investigate, which might be useful to elaborate on the in vivo effect of icaritin.

## 3. Material and Methods

### 3.1. Materials

Laminin, polyornithine, 3,3,5-Triiodo-l-thyronine (T3), paraformaldehyde (PFA), bis(cyclohexanone), oxaldihydrazone (Cuprizone, CPZ), Hoechst 33342 and Bovine Serum Albumin (BSA) were purchased from Roche (Basel, Switzerland). *PDGFaa* was purchased from Peprotech (Cranbury, NJ, USA). Fulvestrant (FV), sidenafil (SDNF), clozapine (CLZ), clemastine (CLN), donepezil (DNPZ), LY294002 and SB203580 were bought from Med Chem Express (Monmouth Junction, NJ, USA). Corticosrone (CORT) and glutamate (Glu) were from Sigma-Aldrich (St. Louis., MO, USA).

Emodin, genipin, icaritin (ICT), icariin (ICA), quercetin, apigenin, loganin and morroniside were purchased from Chengdu Herbpurify Co., Ltd., (Chengdu, China), Resveratrol was bought from JF-NATURAL (Tianjin, China), Longistyline and curcumin (CUR) were purchased from National Institute for the Control of Pharmaceutical and Biological Products (NICPBP, Beijing, China), Longistyline A and 1226 were kindly gifted from Prof. Ruile Pan in the Institute of Medicinal Plant Development (IMPLAD, Beijing, China), B4 was gifted from the Institute of Materia Medica Chinese Academy of Medical Science (IMM, Beijing, China).

Primary antibodies: mouse monoclonal anti-Glial Fibrillary Acidic Protein (GFAP), rabbit polyclonal anti-Microtubule Associated Protein 2 (MAP2), anti-Galc, mouse monoclonal anti-CNPase (CNPase), anti-A2B5 (A2B5), mouse monoclonal anti-O4 (O4) were bought from Millipore (Burlington, MA, USA), anti-Olig2 was from R&D (Minneapolis, MN, USA); anti-MOG was obtained from Santa Cruz (Dallas, TX, USA), PDGFRα and mouse monoclonal anti-Myelin Basic Protein (MBP) were from Abcam (Cambridge, UK). Secondary antibodies: Alexa Fluor 488 Goat anti-mouse IgG, Alexa Fluor 568 Goat anti-mouse IgM, Alexa Fluor 488 Goat anti-rabbit IgG and Alexa Fluor 555 Goat anti-rabbit IgG were bought from Thermo Fisher Scientific (Waltham, MA, USA). AchE, ChAT and Ach ELISA kits were from Gelatins^®^ (Shanghai Crystal Antibiotic Engineering Co., Ltd., Shanghai, China). Cell Counting Kit-8 and BCA protein kit were bought from BioDee Biotechnology (Beijing BioDee Biotechnology Co., Ltd., Beijing, China). Cell Counting Kit-8 and BCA protein kit were bought from BioDee Biotechnology (Beijing BioDee Biotechnology Co., Ltd., Beijing, China). Trizol was obtained from Takara (Takara Biomedical Technology Co., Ltd., Dalian, China).

### 3.2. Cell Line

The mice neural progenitor cells (mNPCs) were generated from a mouse pluripotent stem cell line derived from 129 × C57 mice (a kind gift from Dr. Sen Wu, China Agricultural University, Beijing, China). mNPCs were differentiated from iPSC using our previous method [20]. Cells in the logarithmic phase were used.

### 3.3. Effects of TCM on Oligodendrocyte Precursor Cells Viability and Differentiation

#### 3.3.1. Effects of Herbs on Cell Viability of Oligodendrocyte Precursor Cells

The MTT method was used to test the effects of ethanol extractums of kidney-tonifying herbs on the cell viability of mice neural progenitor cells. Neural progenitor cells were seeded into 96-well plates at a density of 1.2 × 10^4^ cells/well and incubated for attachment. Ethanol was removed from ethanol extractums of kidney-tonifying herbs by vacuum distillation, extracts were dissolved in DMSO at high concentrations and diluted with a cell culture medium before use. Extractums at a series of concentrations were added into wells and co-incubated for 24 h. Cells without treatment were used as control. Absorbance at a wavelength of 570 nm was measured using a microplate reader (FLUOstar Optima, BMG LABTECH, Ortenberg, Germany). The cell viability was calculated by comparing absorbance with control. Doses with cell viability higher than 80% were regarded as non-toxic and used in subsequent studies.

#### 3.3.2. Effects of Herbal Monomers on Differentiation

Neural progenitor cells were seeded into 96-well plates at a density of 1.5 × 10^5^ cells/well (200 μL) and incubated for attachment. The medium was replaced with herbal monomer-containing medium, followed by 72-h incubation. Cells treated with DNPZ were used as controls [16]. The expression of A2B5 was analyzed with immunofluorescence, and the increased expression of A2B5 indicated enhanced differentiation from neural progenitor cells toward oligodendrocyte precursor cells. The quantification of A2B5 intensity used the same method as our previous study [20].

The expression of the MBP protein was used to evaluate the differentiation towards oligodendrocytes. Oligodendrocyte precursor cells were seeded into 96-well plates at a density of 1.5 × 10^5^ cells/well (200 μL) and incubated for attachment. The medium was discarded, and oligodendrocyte culture medium (without T3) with monomers was added and incubated for 72 h. Medium with or without T3 was used as a positive and negative control, respectively. Monomers with elevated expressions of MBP were regarded as promoters of oligodendrocyte precursor cell differentiation.

The effects of icaritin on the differentiation of oligodendrocyte precursor cells were assessed using immunofluorescent staining. Fulvestrant, clemastine, clozapine, donepezil, and sildenafil were used as controls. Briefly, 5 M of control or icaritin was incubated for 72 h with neural progenitor cells or oligodendrocyte precursor cells after attachment. The markers A2B5 and MBP were stained to assess the differentiation of neural progenitor cells and oligodendrocyte precursor cells [20]. Briefly, a threshold was set for each channel (red, green, and blue) and applied to all images with ImageJ software (version 2.0.0, National Institute of Health (NIH), Bethesda, MD, USA) to test if the positive pixels were representative. Then the number of positive pixels was counted, and the data were presented as a relative expression (% of DAPI). The number of red or green positive pixels was divided by the number of blue positive pixels.

Markers, such as MAP2, O4, GlaC, GFAP, CNPase, and MBP, were stained for cell recognition, whereas GFAP^+^ and MAP2^+^ were for identifications of astrocytes and neurons, respectively. O4^+^, Olig2^+^, Galc^+^, MOG^+^, CNPase^+^, GFAP^−^ for oligodendrocytes.

#### 3.3.3. Effects of Herbal Monomers on Cell Viability under Pathological Conditions In Vitro

Pathological models were developed with Glu or CORT. Neural progenitor cells were seeded in a 96-well plate at a density of 1.5 × 10^5^/well and incubated for attachment. The medium was replaced with Glu or CORT-containing medium and co-incubated for 48 h. Cells treated with DMSO were used as controls. Cell viability was measured with CCK-8 according to the manufacturer’s instructions. Concentrations with a cell viability of ~ 80% were used for pathological model development.

The effects of monomers on the cell viability of oligodendrocyte precursor cells under pathological conditions (500 μM of Glu and 100 μM of CORT-induced pathological conditions) were tested. Cells were incubated with CORT, Glu, and monomers at different concentrations for 48 h. DNPZ at concentrations of 20 and 100 μM was used as a positive control, and cells without treatment were negative controls. Cell viability was calculated from the absorbance at a wavelength of 450 nm.

#### 3.3.4. Effects of Icaritin on Differentiation under Pathological Models In Vitro

The effects of icaritin on neural progenitor cell differentiation were assessed using a 3D hydrogel model [20]. 3D matrix, as an in vitro tool, is suitable for long-term culture and can imitate the physical scaffolds and mimicking the niche. It is ideal for exploring the in vivo fate of neural progenitor cells under pathological conditions. Neural progenitor cells in hydrogel were co-incubated with DMSO (control), CORT, or CORT + icaritin (5 μM), respectively. The medium was replaced every three days. On Day 30 and Day 60, cells were washed with PBS three times (5 min per time) and then fixed with paraformaldehyde (4%, pH 7.0) for 1 h at room temperature (RT). The hydrogels were cryoprotected with sucrose solution (30% in PBS) at 4 °C overnight after three rinses with PBS. The hydrogels were embedded in the O. C. T. compound and cut into sections (12 μm-thickness) using a Leica CM1950 Cryostat (Leica, Germany) and stored at −80 °C until analysis. The quantification of MBP intensity used the same method as our previous study [20].

A2B5, GFAP, and MBP were stained using anti-A2B5, anti-GFAP, and anti-MBP antibodies, respectively. Sections were permeabilized with Triton X-100 (0.25% in PBS) for 10 min and blocked with BSA solution (2%) for 1 h to avoid unspecific bindings. Primary antibody solutions (diluted with BSA containing PBST solution, 1%) were added and incubated overnight at 4 °C, followed by secondary antibody incubation (1:1000, diluted with BSA containing PBST) for 1 h (RT). Secondary antibodies included (Alexa Fluor 488 Goat anti-mouse IgG, Alexa Fluor 568 Goat anti-mouse IgM, Alexa Fluor 488 Goat anti-rabbit IgG, Alexa Fluor 555 Goat anti-mouse IgG, Alexa Fluor 555 Goat anti-rabbit IgG (Thermo Fisher Scientific, USA). DAPI was dropped for nucleus staining. Representative images were taken with a laser confocal microscope (TCS-CS8, Leica, Germany) under identical conditions for inter-comparison within the group. The expressions of biomarkers were presented as the read-out of fluorescence intensity. Results were presented as relative expression (percentage of DAPI, %) by counting the positive pixels compared to the threshold using Image J software. Samples were counted in triplicate using different batches of cells originating from the differentiation procedure [20].

### 3.4. Animal Study

#### 3.4.1. Open Filed and Y-Maze Tests

For animal studies, adult male balb/c mice (7–8 weeks old, Beijing Vital River Laboratory Animal Technology, Beijing, China) were used. All animal studies were approved and monitored by IMPLAD (license no. SYXK 2017-0020). Animals were acclimated for a week before the animal study with free chow and water at the temperature of 22 ± 2 °C (relative humidity: 45 ± 4% with a light/dark cycle of 12 h).

Cuprizone (CPZ) was administered for the development of demyelinating mice [27]. The in vivo experimental schedule is illustrated in Figure 5A. Mice were randomly divided into 8 groups (*n* = 14), including the control group (treated with saline), the model group (treated with cuprizone to induce the demyelination model), the donepezil group (DNPZ, demyelinating mice treated with donepezil, 10 mg/kg), the icariin group (ICA, demyelinating mice treated with ICA, 10 mg/kg), the clozapine group (CLZ, demyelinating mice treated with CLZ, 10 mg/kg), and the icaritin groups (ICT, demyelinating mice treated with ICT at low, medium, and high doses of 5, 10, and 15 mg/kg, respectively). Mice in the control group were normally fed; all the other mice were fed a CPZ-containing diet (0.2%) for 5 weeks, followed by standard feeding for 1 week. Then, mice were given PBS (containing 1% DMSO), DNPZ, ICA, CLZ, or ICT (suspended in DMSO-containing PBS) for successively 7 days through intraperitoneal injection (ip) after the withdrawal of CPZ.

The open field test (OFT) was performed to investigate the behavioral effect of icaritin on demyelinating mice. Four white boxes (100 cm × 100 cm × 40 cm) compose the apparatus. Mice were allowed 5 minutes of free movement in the center of the chamber. The total moving distance and time/distance ratio in the central area were digitally recorded throughout the whole movement with a computer-aided image analysis system for a real-time detection test. Ethanol spray was used to clean the arenas to avoid the potential scent cues left by the former mice.

The short-term spatial memory of demyelinating mice was tested with the spontaneous alteration Y-maze device (ANY-maze animal behavior analysis system. Stoelting, Kiel, WI, USA). Three equal-sized arms distributed a “Y” shape where they interconnected at the angle of 120° within the dimensions of 35 × 8 × 10 cm. Mice were allowed free exploration for 8 min after habituating to the test room for 30 min without seeing the Y-maze. The Y-maze was cleaned with 75% ethanol and dried prior to the next test. The behaviors were automatically recorded with a video camera, and spontaneous alterations were assessed by analyzing the series of entries to the arms of the maze, and calculated with the following formula:

$$\mathrm{Percentage}\;\mathrm{of}\;\mathrm{alteration}\;(\%)=\frac{actual\;alteration}{Total\;number\;of\;arm\;entries-2}\times 100\%\;$$

where enhanced spatial memory was indicative of an increased percentage of alteration.

#### 3.4.2. Expressions of MBP in Hippocampus

Mice were sacrificed on Day 8 after the behavioral tests, and 4 mice were chosen from each group for brain perfusion intracardially with 20–25 mL PBS, followed by 20–25 mL of cold (4 °C) 4% paraformaldehyde (PFA) containing PBS (pH = 7.4). Brain tissues were dissected and fixed in 4% PFA overnight (4 °C), followed by cryoprotection in 15% sucrose overnight at 4 °C and transfer to 30% sucrose overnight (4 °C) when the brain tissues sank to the bottom. Tissues were embedded in an O.C.T. compound Tissue-Tek (Sakura, Torrance, CA, USA) for cryosection (12 μM thickness, 3 sections per mice) with a Leica CM1950 Crystat (Leica, Germany). The immunofluorescence of MBP in the corpus callosum was stained following the protocol of cell immunofluorescence staining using frozen sections. Images were taken under a confocal microscope. Samples were counted in triplicate using tissues from different animals. The intensities of MBP were quantified [20].

Hippocampus was taken on ice immediately after sacrifice (10 mice) and washed three times with cold PBS, frozen in liquid nitrogen for western blot analysis.

### 3.5. Mechanisms

#### 3.5.1. Effects of Icaritin on Ach, ChAT, and AchE

Neural progenitor cells were digested into a single-cell suspension with trypLE and seeded into 12-well plates at a density of 5 × 10^4^ cells/well (1 mL) and incubated overnight for attachment. The medium was replaced with icaritin (5 μM) containing PDGF medium and incubated at 37 °C; cells treated with DMSO were used as controls. After a 48-h incubation, the medium was collected and centrifuged at 5000× *g* for 5 min, and the supernatant was transferred to a new tube and stored at 80 °C until analysis.

After the collection of the medium, cells were digested into a single cell suspension with trypLE, centrifugated, and resuspended in PBS (100 μL per well). The obtained cell suspension was sonicated (2 × 10^6^ Hz for 5 s at an interval of 5 s, repeated 30 times. Proteins were obtained by centrifuging at 12,000× g for 5 min and were quantified using the BCA Protein Assay Kit. Ach, ChAT, and AchE were measured using an ELISA kit according to the manufacturer’s instructions.

#### 3.5.2. Effects of Icaritin on Myelin-Related Genes

Cells were seeded into 96-well plates at a density of 1.5 × 10^5^ cells/well (200 μL) and treated with DMSO, icaritin (5 μM), or DNPZ (5 μM) for 48 h at 37 °C, respectively. Total RNA was extracted after Trizol lysis. The reverse transcription was conducted according to Takara’s instructions. Briefly, reverse transcription reacted on ice by mixing an aliquot 2 μL of Prime Script RT Master Mix (5×) and 8 μL of total RNA (50 ng/μL). The reaction lasted for 15 min at 37 °C followed by 5 min at 85 °C, cNDA was stored at −20 °C when the reaction was completed.

Myelin-related genes, MBP, *SOX10*, *NGN3*, *NKX2.2*, *PDGFaa*, and *Olig2*, were determined using quantitative Real-time PCR ( Bio-Rad Laboratories, Inc., Hercules, CA, USA) according to Takara’s instructions (SYBR Premix EX Taq II, Tli RNaseH Plus). The design of primers was shown in Table 3, and the PCR reaction system was demonstrated in Table 4.

#### 3.5.3. Neuroprotective Effects of Icaritin against Pathological Damage of Myelin Related Pathway

Neural progenitor cells were treated with icaritin (5 μM), LY294002 (inhibitor of PI3K), SB203580 (inhibitor of p38 MAPK), or the combination of icaritin + LY294002 (or icaritin + SB203580) under pathological conditions. Cell viability was determined with CCK-8 after 48-h-incubation.

### 3.6. Statistical Analysis

Results were presented as the mean ± standard error of the mean (SEM), unless otherwise stated. Data analysis was performed using one-way factorial analysis of variance (ANOVA), followed by post hoc comparisons using the Dunnett’s or Student–Newman–Keuls (SNK) tests. A probability value of 95% (*p* < 0.05, marked as an asterisk) was used to assess statistical significance. Graphs were prepared in Prism (GraphPad, version 8.3.0) from the raw data.

## 4. Conclusions

In this study, we found that icaritin promoted myelin formation in multiple stages, including enhancing the viability of oligodendrocyte precursor cells and the differentiation of oligodendrocyte lineage cells under pathological conditions and facilitating the maturation of oligodendrocytes and myelin formation under a simulated pathological condition. Importantly, icaritin improved cognition and the behavioral functions of demyelinating mice in vivo. Basically, our study reveals that icaritin has the potential for promoting myelination.

## Figures and Tables

**Table 1:** Effects of compounds on the percentage of A2B5^+^ oligodendrocyte precursor cells (OPCs, *n* = 3, mean ± SD).

Group	Concentration	A2B5^+^ OPCs (%)
CTRL	/	61.53 ± 1.89
Catalpol	5 μM	70.83 ± 1.60 **
Geniposide		69.20 ± 3.66 *
Emodin		62.44 ± 3.79
Longistyline A		59.35 ± 1.91
Curcumin		66.80 ± 2.91
Resveratrol		68.50 ± 3.24 *
Genipin		65.36 ± 3.78
Icaritin		75.34 ± 2.97 ***
Icariin		66.27 ± 3.53
Quercetin		62.48 ± 2.55
Apigenin		61.95 ± 5.80
Loganin		62.23 ± 2.65
Morroniside		59.01 ± 1.49

* *p* < 0.05; ** *p* < 0.01; *** *p* < 0.001 compared with CTRL group.

**Table 2:** Effects of compounds on the percentage of MBP^+^ oligodendrocytes (OLs, *n* = 3, mean ± SD).

Group	Concentration	MBP^+^ OLs (%)
+T3	60 ng/mL	19.03 ± 1.50 **
−T3	/	11.37 ± 1.84
Catalpol	5 μM	15.47 ± 2.38
Geniposide		14.21 ± 3.17
Emodin		6.05 ± 2.39
Cajanine		10.36 ± 4.52
Longistyline A		5.19 ± 3.85
Curcumin		8.92 ± 3.17
Resveratrol		14.20 ± 3.56
Genipin		12.59 ± 2.44
Icaritin		17.00 ± 1.61 *
Icariin		17.86 ± 1.40 **
Quercetin		9.77 ± 1.58
Apigenin		10.83 ± 3.69
Loganin		7.32 ± 2.11
Morroniside		11.46 ± 2.87

* *p* < 0.05; ** *p* < 0.01 compared with −T3 group. T3: polyornithine, 3,3,5-Triiodo-l-thyronine.

**Table 3:** Primer design for Real-time PCR.

Primer	5′ to 3′
MBP	F: GCTTCTTTAGCGGTGACAGGG
	R: TGGAGGTGGTGTTCGAGGTG
*SOX10*	F: AGCCCAGGTGAAGACAGAGA
	R: CCCCTCTAAGGTCGGGATAG
*NGN3*	F: CTGCGCATAGCGGACCACAGCTTC
	R: CTTCACAAGAAGTCTGAGAACACCAG
*NKX2.2*	F: CTCCAATACTCCCTGCACGG
	R: GATTGTGCTCCCAGGCCTG
*PDGFaa*	F: ACCAGACCCAGACATGGCC
	R: AAGACGGCACAGGTCACCAC
*Olig2*	F: TGGCTTCAAGTCATCTTCCTCC
	R: TCGCTCACCAGTCGCTTCAT

**Table 4:** PCR reaction system.

Reagent	Volume (μL)
SYBR Premix EX Taq II (Tli RNaseH Plus)	12.5
PCR Forward Primer (10 μM)	1
PCR Reverse Primer (10 μM)	1
dDNA	2
dH_2_O	8.5
Total	25

## Data Availability

Data are available upon reasonable request to the corresponding author.

## Supplementary Materials

The following supporting information can be downloaded at: https://www.mdpi.com/article/10.3390/molecules28155837/s1, Figure S1: Non-toxic dose screening of herbal monomers through the MTT method. PPD (A), PPT (B), 1226 (C), Geniposide (D), Curcumin (E), Catalpol (F), B4 (G), Genipin (H), Emodin (I), Loganin (J), Icaritin (K), Icariin (L), Quercetin (M), Apigenin (N), Morroniside (O), Resveratrol (P), Longistyline A (Q), Cajanine (R). Figure S2. PDGFRα^+^ OPCs. Scale bars represent 25 μm. Figure S3. Expression of hOLs markers: (A) GFAP^+^ astrocytes and MAP2^+^ neurons, scale bar 25 μm; (B) O4^+^ and Olig2^+^ OL, scale bar 50 μm; (C) Galc^+^ and MOG^+^ OL, scale bar 25 μm; (D) CNPase^+^ and GFAP^−^ OL, scale bar 25 μm; (E) MBP^+^ and Olig2^+^ OL, scale bar 50 μm. Table S1: Effects of TCM constituents on mNPCs viability under Glu-induced pathological condition. (Data are presented as mean ± SD, *n* = 6), Glu: glutamate. Table S2: Effects of TCM on mNPCs viability constituents under CORT-induced pathological condition. (Data are presented as mean ± SD, *n* = 6), CORT: corticosrone.
