# Supplementary material for: Icaritin Promotes Myelination by Simultaneously Enhancing the Proliferation and Differentiation of Oligodendrocyte Precursor Cells
Source: Molecules. 2023 Aug 3;28(15):5837. doi: 10.3390/molecules28155837 (PMC10421464; doi:10.3390/molecules28155837)
Supplement: Supplementary file 1 [file molecules-28-05837-s001.zip › molecules-2458212-suppl.pdf]

Supplementary Information

Icaritin Promotes Myelination by Simultaneously Enhancing the Proliferation and Differentiation of Oligodendrocyte Precursor Cells

Feifei Yang [1], Han Wen [1], Siqi Ma [1], Qi Chang [1], Ruile Pan [1], Xinmin Liu [2] and Yonghong Liao [1,*]

[1] Key Laboratory of Bioactive Substances and Resources Utilization of Chinese Herbal Medicine (Ministry of Education), Institute of Medicinal Plant Development (IMPLAD), Chinese Academy of Medical Sciences & Peking Union Medical College, No. 151 Malianwa North Road, Haidian District, Beijing 100193, China
[2] Institute of Drug Discovery Technology, Ningbo University, No. 818 Fenghua Road, Ningbo 315211, China
* Correspondence: yhliao@implad.ac.cn; Tel./Fax: +86-010-57833268

Material and Methods (catalog no for antibodies)

Primary antibodies: mouse monoclonal anti-Glial Fibrillary Acidic Protein (GFAP, Millipore (Burlington, MA, USA), cat. no. G6171), rabbit polyclonal anti-Microtubule Associated Protein 2 (MAP2, Millipore, cat. no. AB2290A4), Anti-Galc (Millipore, cat. no. MAB342), mouse monoclonal anti-CNPase (Millipore, cat. no. c5922), Anti-A2B5 (Millipore, cat. no. MAB312), mouse monoclonal anti-O4 (Millipore, cat. no. O7139), mouse monoclonal anti-Myelin Basic Protein (anti-MBP, Abcam (Cambridge, UK), cat. no. ab62631), PDGFRα (Abcam, cat. no. ab203491), Anti-Olig2 (R&D (Minneapolis, MN, USA), cat. no. AF2418) and Anti-MOG (Santa Cruz (Dallas, TX, USA), cat. no. sc-376138). Secondary antibody: Alexa Fluor 488 Goat anti-mouse IgG (Thermo Fisher Scientific, Waltham, MA, USA. cat. no. A-11001), Alexa Fluor 568 Goat anti-mouse IgM (Thermo Fisher Scientific, USA. cat. no. A-21043), Alexa Fluor 488 Goat anti-rabbit IgG (Thermo Fisher Scientific, USA, cat. no. A-11008), and Alexa Fluor 555 Goat anti-rabbit IgG (Thermo Fisher Scientific, USA. cat. no. A-21428).

Supplementary Figures

Figure S1 Non-toxic dose screening of herbal monomers through MTT method. PPD (A), PPT (B), 1226 (C), Geniposide (D), Curcumin (E), Catalpol (F), B4(G), Genipin (H), Emodin (I), Loganin (J), Icaritin (K), Icariin (L), Quercetin (M), Apigenin (N), Morroniside (O), Resveratrol (P), Longistyline A (Q), Cajanine (R).

Figure S2 PDGFRα^{+} positive OPCs (human). Scale bars represent 25 μm.

Figure S3. Expression of hOLs markers: (A) GFAP^{+} astrocytes and MAP2^{+} neurons, scale bar 25 μm; (B) O4^{+} and Olig2^{+} OL, scale bar 50 μm; (C) Galc^{+} and MOG^{+} OL, scale bar 25 μm; (D) CNPase^{+} and GFAP- OL, scale bar 25 μm; (E) MBP^{+} and Olig2^{+} OL, scale bar 50 μm.

Figure S1

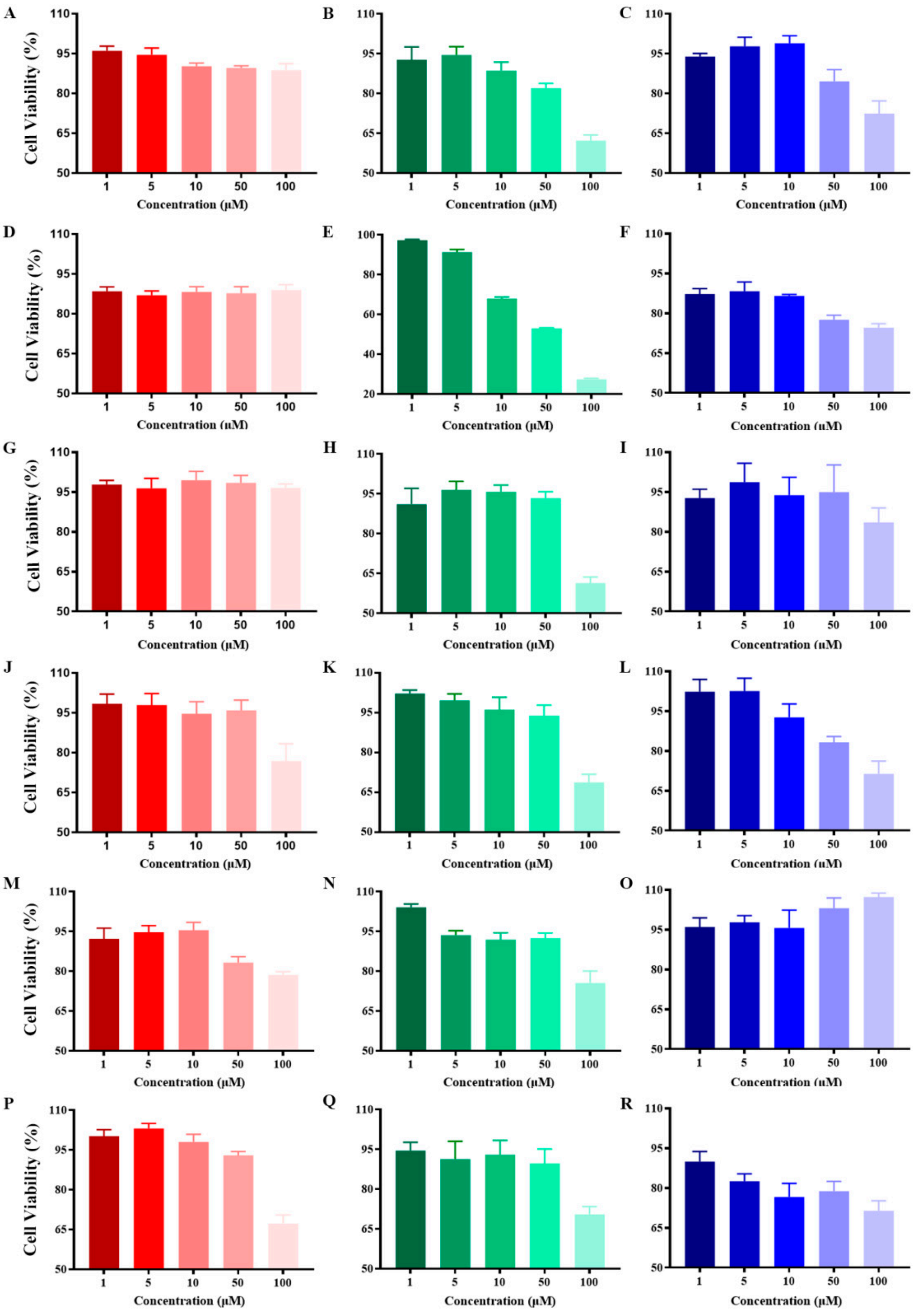
A
B
C
D
E
F
G
H
I
J
K
L
M
N
O
P
Q
R
Cell Viability (%)
110
95
80
65
50
100
80
60
40
20
1
5
10
50
100
Concentration (μM)

Figure S2.

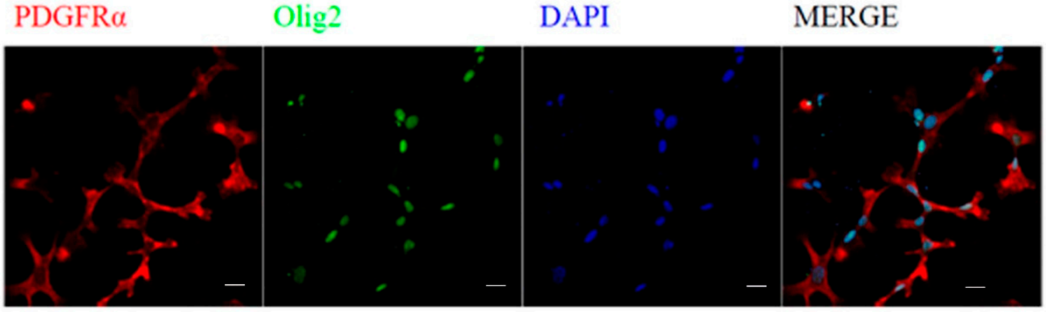

Figure S3

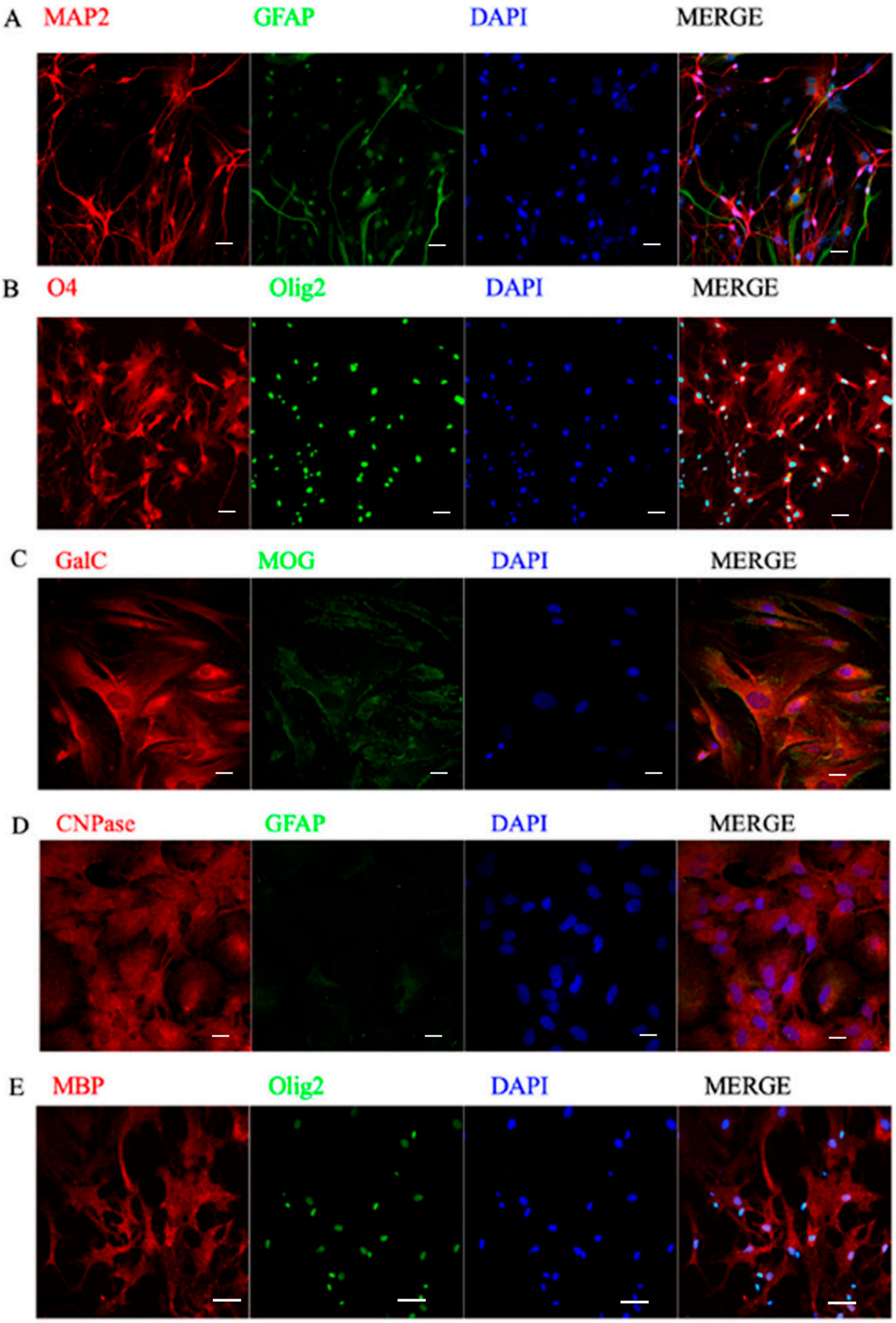
A
MAP2
GFAP
DAPI
MERGE
B
O4
Olig2
DAPI
MERGE
C
GalC
MOG
DAPI
MERGE
D
CNPase
GFAP
DAPI
MERGE
E
MBP
Olig2
DAPI
MERGE

Supplementary Tables:

Table S1. Effects of TCM constituents on Glu-induced reduction viability of mNPCs. (Data are presented as mean ± SD, n=6), Glu: glutamate.

Table S2. Effect of TCM constituents on CORT-induced reduction viability of mNPCs. (Data are presented as mean ± SD, n=6), CORT: corticosrone.

Table S1 Effects of TCM constituents on mNPCs under Glu-induced pathological condition (Data are presented as mean ± SD, n=6)

Group	Concentration	Cell viability (%)
CTRL	/	100.23±1.82
Glu	500 μM	81.56±2.23
DNPZ	20 μM	75.82±1.37
	100 μM	48.67±3.49
PPT	2 μM	82.59±4.92
	10 μM	78.45±3.27
	50 μM	70.33±3.96
PPD	2 μM	80.98±4.25
	10 μM	75.32±3.97
	50 μM	61.23±3.44
1226	2 μg/mL	72.49±3.75
	10 μg/mL	68.43±2.66
	50 μg/mL	55.43±4.94
Catalpol (CAT)	2 μM	82.25±3.76
	10 μM	83.51±4.75
	50 μM	88.66±1.53
Geniposide	2 μM	82.73±3.24
	10 μM	81.52±2.24
	50 μM	75.43±4.97
Emodin	2 μM	77.33±5.96
	10 μM	65.72±4.28
	50 μM	40.42±2.15
Cajanine	2 μM	77.25±4.36
	10 μM	49.51±3.27
	50 μM	36.95±1.48
Longistyline A (LoA)	2 μM	91.32±1.76
	10 μM	79.34±5.28
	50 μM	52.38±2.40
B4	2 μg/mL	89.77±2.37
	10 μg/mL	94.35±1.92
	50 μg/mL	76.92±2.51

	2 μM	92.84±1.86
Curcumin (CUR)	10 μM	98.72±1.40
	50 μM	64.59±3.82
	2 μM	80.32±4.58
Resveratrol	10 μM	79.40±2.14
	50 μM	72.83±4.59
	2 μM	83.10±2.59
Genipin (GEN)	10 μM	90.26±0.84
	50 μM	75.25±3.83
	2 μM	84.59±2.40
Icaritin (ICT)	10 μM	115.56±1.79
	50 μM	123.02±1.64
	2 μM	72.39±4.30
Icariin	10 μM	74.66±2.29
	50 μM	63.14±3.71
	2 μM	82.42±3.72
Quercetin	10 μM	72.03±0.71
	50 μM	55.23±3.93
	2 μM	84.38±3.56
Apigenin	10 μM	66.95±3.25
	50 μM	66.38±4.58
	2 μM	82.92±2.96
Loganin	10 μM	80.50±3.83
	50 μM	79.31±2.12
	2 μM	85.49±2.25
Morroniside	10 μM	79.56±3.01
	50 μM	79.31±2.29

Table S2 Effects of TCM on mNPCs constituents under CORT-induced pathological condition

(Data are presented as mean ± SD, n=6)

Group	Concentration	Cell viability (%)
CTRL	/	98.29±2.52
CORT	100 μM	67.01±1.86
DNPZ	20 μM	65.82±1.73
	100μM	63.59±2.78
PPT	2 μM	68.77±3.21
	10 μM	70.24±3.25
	50 μM	55.78±3.52
PPD	2 μM	59.32±4.51
	10 μM	64.59±2.18
	50 μM	53.47±3.86
1226	2 μg/mL	68.34±2.71
	10 μg/mL	57.59±3.50
	50 μg/mL	53.24±4.67
Catalpol (CAT)	2 μM	69.16±3.82
	10 μM	71.23±2.56
	50 μM	76.90±1.54
Geniposide	2 μM	70.12±3.25
	10 μM	64.87±2.14
	50 μM	60.74±3.89
Emodin	2 μM	63.27±1.82
	10 μM	61.90±2.78
	50 μM	52.34±3.25
Cajanine	2 μM	62.18±3.42
	10 μM	59.63±1.39
	50 μM	38.72±2.91
Longistyline A	2 μM	63.58±3.46
	10 μM	59.10±2.17
	50 μM	36.54±1.35
B4	2 μg/mL	69.36±2.47
	10 μg/mL	68.12±1.79
	50 μg/mL	68.45±2.36

Group	Concentration	Cell viability (%)
Curcumin (CUR)	2 μM	73.67±1.71
	10 μM	75.82±1.55
	50 μM	42.35±3.68
Resveratrol	2 μM	66.38±2.37
	10 μM	59.48±3.32
	50 μM	52.31±2.90
Genipin (GEN)	2 μM	71.2±3.84
	10 μM	75.21±1.74
	50 μM	78.32±1.99
Icaritin (ICT)	2 μM	70.45±1.95
	10 μM	92.43±3.62
	50 μM	100.30±2.15
Icariin (ICA)	2 μM	71.04±3.91
	10 μM	69.85±2.76
	50 μM	62.31±2.32
Quercetin	2 μM	71.03±3.63
	10 μM	42.99±2.64
	50 μM	38.78±3.20
Apigenin	2 μM	72.24±3.10
	10 μM	69.72±3.09
	50 μM	44.98±2.20
Loganin	2 μM	71.41±2.53
	10 μM	72.03±2.11
	50 μM	71.85±2.11
Morroniside	2 μM	70.37±2.25
	10 μM	72.07±2.70
	50 μM	71.51±3.81
